# The effect of hypericum perforatum on kidney ischemia/reperfusion damage

**DOI:** 10.1080/0886022X.2017.1287734

**Published:** 2017-02-16

**Authors:** Murat Cakir, Halil Duzova, Işil Baysal, Cemile Ceren Gül, Gülbahar Kuşcu, Fatma Kutluk, Hilal Çakin, Şifanur Şeker, Esranur İlbeği, Seda Uslu, Umut Avci, Samet Demir, Cihan Akinci, Sercan Atli

**Affiliations:** a Department of Physiology, Faculty of Medicine, Inonu University, Malatya, Turkey;; b Department of Histology and Embryology, Faculty of Medicine, Inonu University, Malatya, Turkey;; c Faculty of Medicine, Inonu University, Malatya, Turkey

**Keywords:** Kidney, ischemia-reperfusion, hypericum perforatum, oxidative stress, rat

## Abstract

It has been revealed in recent studies that Hypericum Perforatum (HP) is influential on cancer, inflammatory diseases, bacterial and viral diseases, and has neuroprotective and antioxidant properties. In this study, we investigated the effect of HP, which is known to have antioxidant and anti-inflammatory effects, on kidney I/R damage. Male Sprague–Dawley rats were divided into three groups, and each of the groups had eight rats: The Control Group; the Ischemia/Reperfusion (I/R) Group; and the IR + HP Group which was treated with 50 mg/kg of HP. The right kidneys of the rats were removed, and the left kidney developed ischemia during the 45th min, and reperfusion occurred in the following 3rd h. The histopathological findings and also the level of Malondialdehyde (MDA), Glutathione (GSH) and superoxide dismutase (SOD), catalase (CAT), and glutathione peroxidase (GSH-PX) enzyme activations in the renal tissues were measured. Blood Urea Nitrogen (BUN), Creatinin (Cre) from serum samples were determined. The levels of BUN, Cre, and kidney tissue MDA increased at a significant level, and the SOD, CAT, and GSH-PX enzyme activity decreased at a significant level in the I/R group, compared with the Control Group (*p* < 0.05). In the I/R + HP group, the levels of MDA decreased at a significant level compared to the I/R group, while the SOD, CAT, and GSH-PX activity increased (*p* < 0.05). In histopathological examinations, it was observed that the tubular dilatation and epithelial desquamation regressed in the IR + HP Group when compared with the I/R Group. It has been shown with the histological and biochemical results in this study that HP is protective against acute renal I/R.

## Introduction

Kidney Ischemia/Reperfusion (I/R) Damage is a common clinical problem that has high mortality and morbidity rates, and results in Acute Renal Failure (ARF).^1^^,^^2^ During the interventions applied to the kidneys like kidney transplantation and coronary bypass, the blood-flow to the kidneys may be cut off. The cutoff of the blood leads to damages in the tissues. During the reperfusion in which the blood-flow is established again, the increase in the production of free radicals and inflammatory response may result in more damage.^3^ These reactions, which occur after I/R, may cause a process that may proceed as bad as necrosis and apoptosis in kidney tubules.^4^ There are antioxidant defense systems that eliminate the Reactive Oxygen Species (ROS) that are produced in the tissues. Superoxide dismutase (SOD), glutathione peroxidase (GSH-PX), and catalase (CAT), which exists in tissues, remove the ROS in a rapid manner under normal circumstances.^5^ During the I/R, the antioxidant defense system becomes inadequate, and as a result, oxidative damage occurs in the tissues.^6^ These events result in cell damage, which is caused by lipid peroxidation. Oxygen-centered free radicals include Hydroxyl Radical (OH), Hydrogen Peroxide (H_2_O_2_) and the Superoxide Radical (O_2_^•^) and are among well-known radical species that are relevant to reperfusion injury at the highest rate.^7^

Hypericum Perforatum (HP), which is also known as St. John’s Wort (SJW), is a plant that has been used as a medication for 2000 years. It has spread widely in the world in mild-climatic areas of Asia, Europe, America, and Australia.^8^ In studies conducted in recent years, it has been demonstrated that HP is influential on cancer, inflammatory diseases, bacterial, and viral diseases; and has neuroprotective and antioxidant properties.^9^ HP includes bioactive compounds like hypericin and hyperphorin, flavonoids, xanthone derivatives, and biapigenin.^10^ It has been shown in previous studies that HP prevents the apoptosis and DNA damage occurring due to ROS,^11^ and has anti-inflammatory influences.^12^^,^^13^ It has also been demonstrated that it has protective effects like antioxidant and anti-inflammatory effects against intestinal and liver I/R damage.^14^^,^^15^

In this empirical study, we investigated the protective effects of HP, which is used commonly as herbal medication in the world, and which has antioxidant and anti-inflammatory properties, against kidney I/R damage.

## Material and method

### Plant material

Flowering herbs of HP were collected from Malatya (Turkey) in May 2016 and were dried in the shade. The plant was identified by Assistant Prof. Dr. Mustafa Sinan Kaynak and a voucher specimen was stored in the herbarium of İnonu University, Faculty of Pharmacy.

#### Preparation of the plant extract

The dried and coarsely powdered flowering herbs of HP (100 g) were macerated with 80% ethanol (2 × 300 ml) for two days by continuous stirring at room temperature. The extracts were separated from the pellets by filtering twice through the piled filler paper to increase the yield. Extracts adhering to the wall of the glass bowl were separated by an ultrasonic device and were settled to the bottom. Then it was evaporated to dryness under reduced pressure (EtOH extract, yield 38.7 g) by rotary evaporator vacuum (Rotavapor Buchi r-210, Canada). The extract was stored at −80 °C until use in the experiments. The dried extract was dissolved in 10% Tween 80-saline solution before intraperitoneal (IP) administration to animals.

#### Animals

The empirical protocol in this study was approved by the Ethical Committee on Animal Research of Inonu University (permit number 2016 A-05). Twenty-four male Sprague–Dawley rats (*n* = 24), weighing 300–350 g, were obtained from Animals Research Center of Inonu University Laboratory, and were kept in a controlled room. The rats were provided with standard pellet diet and water *ad libitum* at a temperature of 21 ± 2 °C. The light cycle was established as 12:12-h light/dark.

### Experimental protocols

The rats were divided into three groups in random order. In order to anaesthetize/analgesia the animals before surgical operation, Ketamine (Ketalar, Eczacibasi, Istanbul, Turkey) 70 mg/kg and xylazine (Rompun, Bayer, Istanbul, Turkey) 8 mg/kg IP were used. The lumbar areas of the rats were shaved and sterilized with povidine iodine solution. Then midline laparotomies were carried out and right nephrectomy was performed on all the rats in the groups.

Group 1 rats (The Control Group) (*n* = 8), underwent right nephrectomy. After 3 h, the animals were sacrificed by removing the blood from their hearts.

Group 2 rats (The I/R Group) (*n* = 8), underwent right nephrectomy. Then, the left renal artery was occluded with a clamp for 45 min followed by reperfusion for 3 h.

Group 3 rats (The I/R + HP Group) (*n* = 8), underwent the same surgical procedure as in the I/R Group. About 50 mg/kg HP extract was given IP at the beginning of ischemia.

Then, the rats were sacrificed by removing the blood from their hearts, and the left kidneys were removed in a fast manner, decapsulated, and divided into two equal longitudinal parts. One of these parts was placed in formaldehyde solution for histopathological examinations with light microscopy. The other part of the kidney was kept in deep freezer at −80 °C until assayed for Malondialdehyde (MDA), Glutathione (GSH), SOD, CAT and GSH-PX enzyme activity. The blood obtained was separated to evaluate serum levels of Blood Urea Nitrogen (BUN) and Creatinin (Cre) by using an Olympus Autoanalyzer (Olympus Instruments, Tokyo, Japan).

### Glutathione measurement

The Glutathione measurement was made in accordance with the Ellman Method in the homogenates.^16^ The homogenates were mixed with 10% trichloroacetic acid solution in an equal amount, and were centrifuged at 4000 rpm for 10 min. By doing so, an extract that was separated from proteins was obtained. Disodium phosphate solution was added to the non-protein extract. Then, the solution, which was prepared with 5.5-dithiobis-2-nitrobenzoic acid, which itself was dissolved in 1% sodium citrate solution, was added. It was read in the spectrophotometer at 410 nm wavelength and the values were recorded. The calculations were made according to the standard graphics that formed the GSH level.

### Malondialdehyde (MDA) assay

MDA was measured according to the directions set in the method by Uchiyama and Mihara.^17^ The MDA contents of the homogenates were determined as an indicator of lipid peroxidation by monitoring Thiobarbituric Acid (TBA) reactive substance formation. The absorbance was measured by spectrophotometer (UV-1601; Shimadzu, Kyoto, Japan) at 532 and 520 nm. The results were given in nmol/g tissue according to a standard graphic.

### Determination of SOD activity

Total SOD activity was determined according to the directions set in the method by Sun et al.^18^ The principle of the method is the inhibition of Nitro Blue Tetrazolium (NBT) reduction by the xanthine–xanthine oxidase system as a superoxide generator. SOD enzyme activity in the medium creates blue color that is inversely proportional to NBT. The absorbance was measured spectrophotometrically at 560 nm. SOD activity was expressed in U/g protein.

### Determination of CAT activity

CAT enzyme reveals H_2_O_2,_ by converting it into water and oxygen. CAT activity was determined according to the directions set in the method by Aebi.^19^ The method is based on the decomposition of H_2_O_2_ (which is added to the medium by CAT enzyme) and the measurement of the rate of the decrease in the absorbance per unit time. The results were expressed in k/g protein.

### Determination of GSH-PX activity

The GSH-PX activity was measured according to the directions set in the method by Pagia and Valentine.^20^ GSH-PX is an enzyme that converts H_2_O_2_ into water (which is mediated by the oxidation of the reduced glutathione). The reduction of oxidized glutathione requires the oxidization of glutathione reductase and nicotine amide adenine dinucleotide phosphate (NADP), which occurs with NADPH in the medium. Spectrophotometric measurement of the change in the absorbance that occur by adding H_2_O_2_ to the medium containing NADPH, reduced glutathione, sodium azide, and glutathione reductase at 340 nm reflects the GSH-PX activity. This activity was expressed as U/mg protein.

### Protein measurement

The determination of the protein from the supernatant was made according to the directions set in the Lowry Method.^21^ Standard bovine serum albumin was used, and the calibration curve was prepared. The results were expressed as μg/mL.

### Histological evaluation

The kidney samples were kept in 10% formalin and were embedded in paraffin to conduct light microscopic evaluations. The samples that were embedded in paraffin were cut into 5 μm-thick sections. They were then mounted on the slides and stained with Hematoxylin–Eosin (H–E). The tissue samples were examined by using a Leica DFC280 light microscope and a Leica Q Win Image Analysis System (Leica Micros Imaging Solutions Ltd., Cambridge, UK). The sections were examined in terms of tubular dilatation, epithelial desquamation, hydropic changes, and intertubular congestion. The changes in the histologic parameters were determined as 1 (25%), 2 (50%), 3 (50%–75%), and 4 (>75%) according to their spread in the area.

### Statistical analyses

The statistical analyses were made by using SPSS17.0 (SPSS Inc., Chicago, IL, USA). All the groups in the study were compared by the nonparametric Kruskal–Wallis test. The *p* < 0.05 value was considered as being statistically significant. All the results were expressed as Means ± Standard Deviation (SD), and the quantitative data were described as Mean ± Standard Deviation (SD). The normality of the quantitative data was determined with Shapiro–Wilk test; and the Kruskal–Wallis test was made use of to test the study data. The Bonferroni–Corrected Mann–Whitney U test was made use of in pairwise comparisons among all the study groups.

## Results

### The effects of HP on kidney tissue MDA, GSH level, CAT, SOD, GSH-PX enzyme activity, serum BUN, and cre level

As it is observed in Table 1, the MDA level in the I/R Group was higher than the Control and I/R + HP Groups (*p* < 0.05). The tissue CAT, SOD, and GSH-PX enzyme activity in the I/R Group was lower than the Control Group (*p* < 0.05). The increase in the CAT, SOD, and GSH-PX activity in the Groups of I/R + HP was significant when compared with the I/R Group (*p* < 0.05). The serum levels of BUN and Cre were significantly higher in the I/R and I/R + HP Group when compared with the Control Group (*p* < 0.05).

**Table 1.** The levels of MDA, GSH and CAT, SOD, GSH-PX enzyme activity in kidney tissue and the serum levels of BUN, Cre.

Group	MDA (nmol/g tissue)Mean ± SD	GSH (mmol/g) Mean ± SD	CAT (k/g protein) Mean ± SD	SOD (U/mgprotein) Mean ± SD	GSH-PX (U/mg protein) Mean ± SD	BUN (mg/dL) Mean ± SD	Cre (mg/dL) Mean ± SD
Control	238.7 **±**26^a^	122.3 **±**21.4^a^	0.4915 **±**0.032^a^	0.2422 **±**0.03^a^	186.9 **±**7.7^a^	27.59 **±**2.6^a^	0.6150 **±**0.329^a^
I/R	317.5 **±**41^b^	109.1 **±**31.3^a^	0.3712 **±**0.059^b^	0.1533 **±**0.027^b^	165.4 **±**10^b^	31.85 **±**4.8^b^	0.8200 **±**0.124^b^
I/R + HP	249.3 **±**88^a^	134.1 **±**40.6^a^	0.5145 **±**0.053^a^	0.2125 **±**0.024^a^	180.6 **±**6.6^a^	36.3 **±**3.5^b^	0.8937 **±**0.084^b^

a and b are different from each other, ^a,b^*p* < 0.05.

### Histological examination

**Figure 1.**
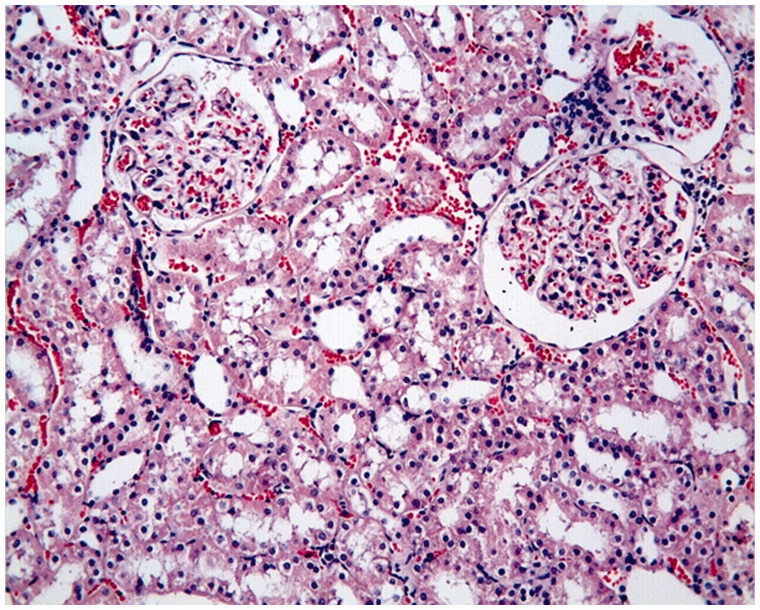
Control group: proximal tubules, distal tubules and glomerular structures are observed in normal histological state, H–E; ×20.

**Figure 2.**
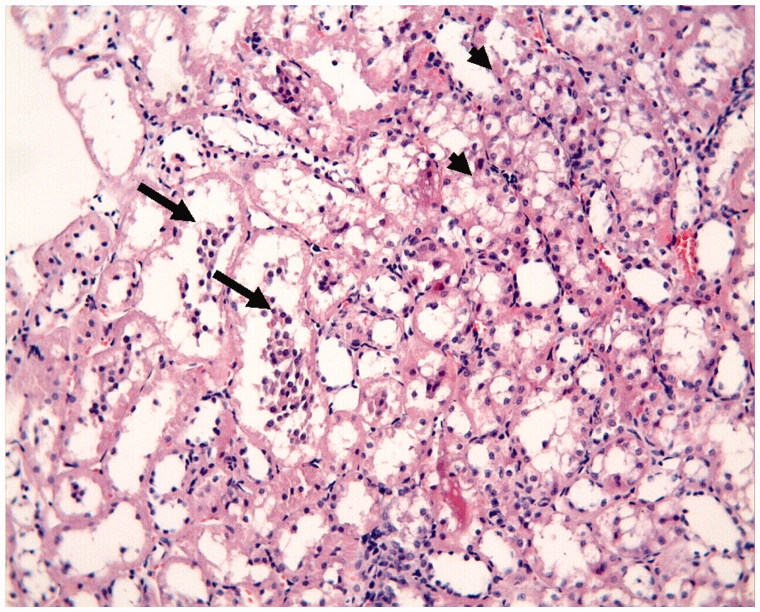
I/R group: degeneration of tubular epithelium (hydropic degeneration (small arrow) in the tubules and cell desquamation (great arrow), H–E; ×20.

**Figure 3.**
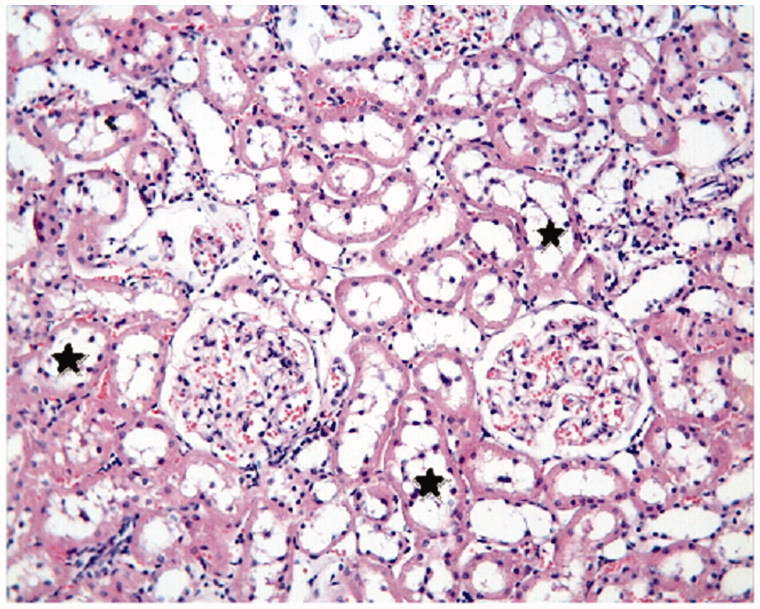
I/R group: tubular dilatation (*), H–E; ×20.

**Figure 4.**
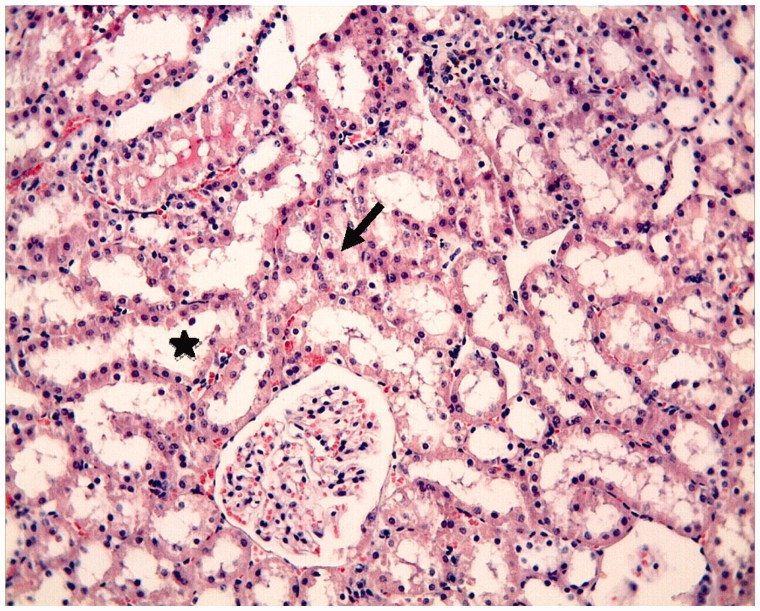
I/R + HP group: tubular dilatation (*) and tubular desquamation (arrow) are observed as being regressed when compared with the I/R group, H–E; ×20.

**Figure 5.**
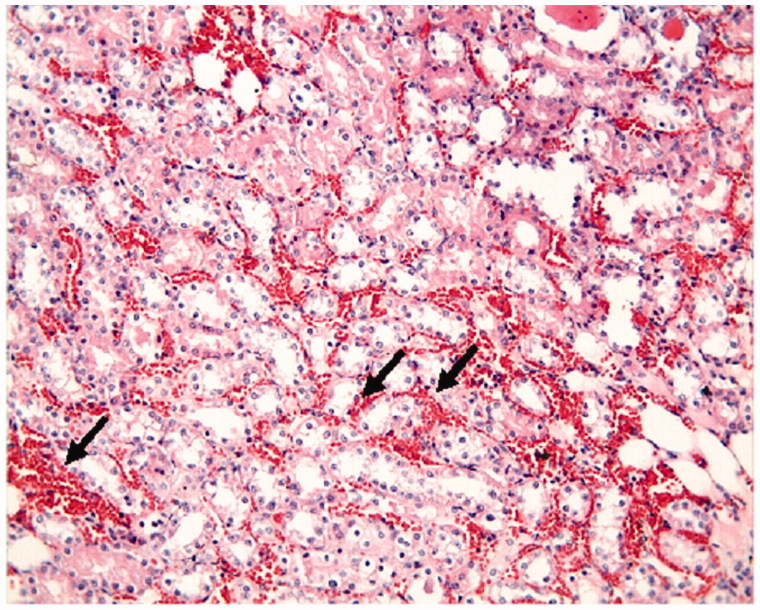
I/R + HP group: intertubular congestion obvious (arrow), H–E; ×20.

The proximal and distal tubules were observed to be natural in the Control Group (Figure 1). On the other hand, tubular epithelial desquamation, tubular dilatation, and hydropic changes were observed in the I/R Group (Figures 2 and 3). It was observed that the tubular dilatation and epithelial desquamation regressed in the IR + HP Group when compared with the IR Group (Figure 4). The most prominent finding in the IR + HP Group was the observing of obvious congestion areas among the tubules (Figure 5). The histopathological score of kidney tissue among the groups is shown in Table 2.

## Discussion

The blood-flow may be cut off temporarily during urologic and vascular events like kidney transplantation and in operations in which the aorta or the renal pedicule is clamped.^22^ Reestablishing the blood flow in the ischemic tissue is a situation that may increase the damage in the tissue, which is a paradoxical situation.^23^ The kidney I/R is a dynamic process in which both the inflammation and some of the mediators that cause the inflammation influence each other. The inflammatory reactions happening during the I/R are among the causes of the oxidative stress and lipid peroxidation.^24^ Endothelial damage, on the other hand, causes that the adhesion molecules increase. Then, leukocyte infiltration occurs towards the interstitial gap. Necrosis and fibrosis follow the polarity and brush border losses in the proximal tubules.^25^ Vascular congestion, edema formation, diminished blood-flow have been showed in the corticomedullary junction of the kidney.^26^ In empirical I/R damage studies conducted on rats, although different ischemia and reperfusion durations were applied, it was concluded in some studies that 45 min ischemia and 3 h reperfusion durations were suitable for the application of the empirical model.^3^^,^^6^

The reestablishment of oxygen to the ischemic kidney results in the production of free oxygen radicals. The free oxygen radicals cause lipid peroxidation and increase the damage in the kidney even more.^5^ ROS triggers the cascade of cytokines and chemokines through NF-kB (Nuclear Factor kappa B) activation.^27^ After I/R, the antioxidant defense system is disrupted. The level of the MDA, which is the product of lipid peroxidation, increases, while decreases are observed in SOD, CAT, and GSH-PX enzyme activations.^28^ Similarly, in our study, while the SOD, CAT, and GSH-PX enzyme activation decreased in the I/R Group, the MDA level increased. In the rats in I/R + HP Group, the CAT, SOD, and GSH-PX enzyme activation increased, and the MDA level decreased. These findings show that HP decreases the oxidative stress developing due to I/R. In histological examination, tubular epithelial desquamation, tubular dilatation and hydropic changes were observed in the I/R Group. The tubular epithelial desquamation, tubular dilatation and hydropic changes decreased in the I/R + HP Group; however, tubular congestion was observed.

Hypericum extracts may be important for the treatment of many diseases that are related with free radical production with their antioxidant effects. In addition to its antidepressant activities, HP, in line with popular credence, also possesses anxiolytic, antiviral, wound-healing, antimicrobial, analgesic, and anti-inflammatory effects^29^ HP may be used alone or in combination with the substances that constitute it for treatment purposes.^10^ HP contains hypericin, hyperphorin, ﬂavonoids, and ﬂavonoid derivatives, xanthone derivatives, and biapigenin. These active components show potential antioxidant activity by free radical scavenging, inhibition of lipid peroxidation, activation of signal transducers.^30^^,^^31^ It was determined that lipid peroxidation and cell-death were decreased in rat-cultured hippocampal neurons by ethanol extracts and the ones with flavonol glycosides, flavonol, and biflavone aglycone. The formation of amyloid-induced ROS were decreased by flavonoids in microglia^32^ Dinamarca et al. conducted a study and reported that the hyperphorinin decreased the Aβ-induced neurotoxicity and oxidative damage.^33^

Benedi et al.^34^ conducted another *in vitro* study and showed that the free radical production and lipid peroxidation formed by H_2_O_2_ decreased with HP extract application. In another *in vitro* study, it was reported that the HP application decreased the HO radical production.^35^ The anti-inflammatory and antioxidant properties of HP resulted in its application in many *in vivo* study models. Mozaffari et al. conducted a study and found that HP extracts decreased inflammation and lipid peroxidation in rat irritable bowel syndrome model.^36^ In another study, it was reported that HP application corrected the serum lipid level and oxidative damage in hyperlipidemic rats.^37^

HP has been used in many I/R study models because of its antioxidant and anti-inflammatory effects. De Paola et al.^30^ conducted a study in Splanchnic Artery Occlusion Model, and reported that HP extract decreased the lipid peroxidation and inflammation, and healed histopathological damage. Genovese et al.^38^ conducted a study on rats and reported that applying HP on spinal cord injury decreased inflammation, apoptosis and histologic damage. In another study conducted in Hepatic I/R Model, it was demonstrated that applying HP decreased the inflammatory cytokine level, lipid peroxidation, and histopathological damage.^14^ Bayramoglu et al.^39^ conducted a study and applied 50 mg/kg intraperitonal single-dose HP, which is the same in our study. Similar to the findings obtained in our study, it was shown that HP application decreased MDA level and increased CAT, GSH-PX enzyme activity, thus decereasing liver I/R damage. In our study, we observed that the antioxidant property of HP is more obvious. HP and the compounds that constitute it may be used for treatment purposes because of their antioxidant properties in many diseases in which oxidative stress happens. HP, which has been used widely in the whole world for centuries, is a safe and cheap herbal medication. In this respect, it may be aplied both before and after I/R in clinics in a comfortable manner.

**Table 2.** The histopathological score of kidney tissue among the groups.

	Hydropic changes				Tubular dilation				Tubular desquamation congestion							
Groups	25 (%)	50 (%)	50–75 (%)	>75 (%)	25 (%)	50 (%)	50–75 (%)	>75 (%)	25 (%)	50 (%)	50–75 (%)	>75 (%)	25 (%)	50 (%)	50–75 (%)	>75 (%)
Control	0	0	0	0	7	0	0	0	7	0	0	0	0	0	0	0
I/R	3	1	3	0	2	1	3	1	2	2	3	0	2	0	1	1
I/R + HP	2	0	0	0	1	0	0	1	1	0	0	1	0	0	0	0

## Conclusions

In this empirical study, we found that the HP treatment prevented the oxidative damage that occurred due to I/R in renal I/R model. The antioxidant effects of the HP observed in our study show similarities with those reported in the literature. We consider that the full healing of histological and biochemical symptoms being not observed stems from the shorter reperfusion time.
